# A Network of Cancer Genes with Co-Occurring and Anti-Co-Occurring Mutations

**DOI:** 10.1371/journal.pone.0013180

**Published:** 2010-10-04

**Authors:** Qinghua Cui

**Affiliations:** Department of Biomedical Informatics, Peking University Health Science Center, Beijing, China; University of Glasgow, United Kingdom

## Abstract

Certain cancer genes contribute to tumorigenesis in a manner of either co-occurring or mutually exclusive (anti-co-occurring) mutations; however, the global picture of when, where and how these functional interactions occur remains unclear. This study presents a systems biology approach for this purpose. After applying this method to cancer gene mutation data generated from large-scale and whole genome sequencing of cancer samples, a network of cancer genes with co-occurring and anti-co-occurring mutations was constructed. Analysis of this network revealed that genes with co-occurring mutations prefer direct signaling transductions and that the interaction relations among cancer genes in the network are related with their functional similarity. It was also revealed that genes with co-occurring mutations tend to have similar mutation frequencies, whereas genes with anti-co-occurring mutations tend to have different mutation frequencies. Moreover, genes with more exons tend to have more co-occurring mutations with other genes, and genes having lower local coherent network structures tend to have higher mutation frequency. The network showed two complementary modules that have distinct functions and have different roles in tumorigenesis. This study presented a framework for the analysis of cancer genome sequencing outputs. The presented data and uncovered patterns are helpful for understanding the contribution of gene mutations to tumorigenesis and valuable in the identification of key biomarkers and drug targets for cancer.

## Introduction

Cancer arises through accumulated genetic and epigenetic alternations of somatic cells [1], [2], [3]. In recent years, numerous efforts have been made to identify gene mutations in various human cancers by genome-wide or large-scale sequencing [3], [4], [5], [6], [7], [8], [9]. In fact, these studies have identified thousands of cancer gene mutations, and certain patterns or rules of cancer gene mutations have been uncovered through the analysis of these mutation data [1], [2], [10], [11]. The discovery of mutated genes in human cancers and the patterns behind the mutation data have provided critical insights into the mechanisms underlying cancer formation and development, and have proven helpful for cancer therapy [3], [12]. However, the rapid increase in cancer gene mutation data suggests a high level of complexity related to understanding the tumorigenic process [2]. Given this complexity, more novel methods for the analyses of mutation data are needed for a better understanding of cancer.

Generally, cancer gene mutations do not occur randomly. Mutations of certain cancer genes tend to co-occur (termed co-occurring mutation in this work), suggesting that they may contribute together to tumor formation and development. However, mutations of some other genes occur in a mutually exclusive fashion (termed anti-co-occurring mutation in this work) [8], suggesting that two genes with anti-co-occurring mutation may have highly similar downstream components. For example, Ras and Braf show anti-co-occurring mutations. Indeed, activaion of one member is sufficient for activating the MAPK pathway [13]. Although some cancer genes with co-occurring and anti-co-occurring mutations have been revealed, the complexity of human cancer gene mutations prevents us from gaining a global landscape of cancer gene co-occurring and anti-co-occurring mutations. For better understanding these functional interactions among cancer genes, this study presents a systems biology approach and conducts a comprehensive analysis of cancer genes with co-occurring mutations and anti-co-occurring mutations.

## Results

### Network of co-occurring and anti-co-occurring cancer gene mutations

**Figure 1:**
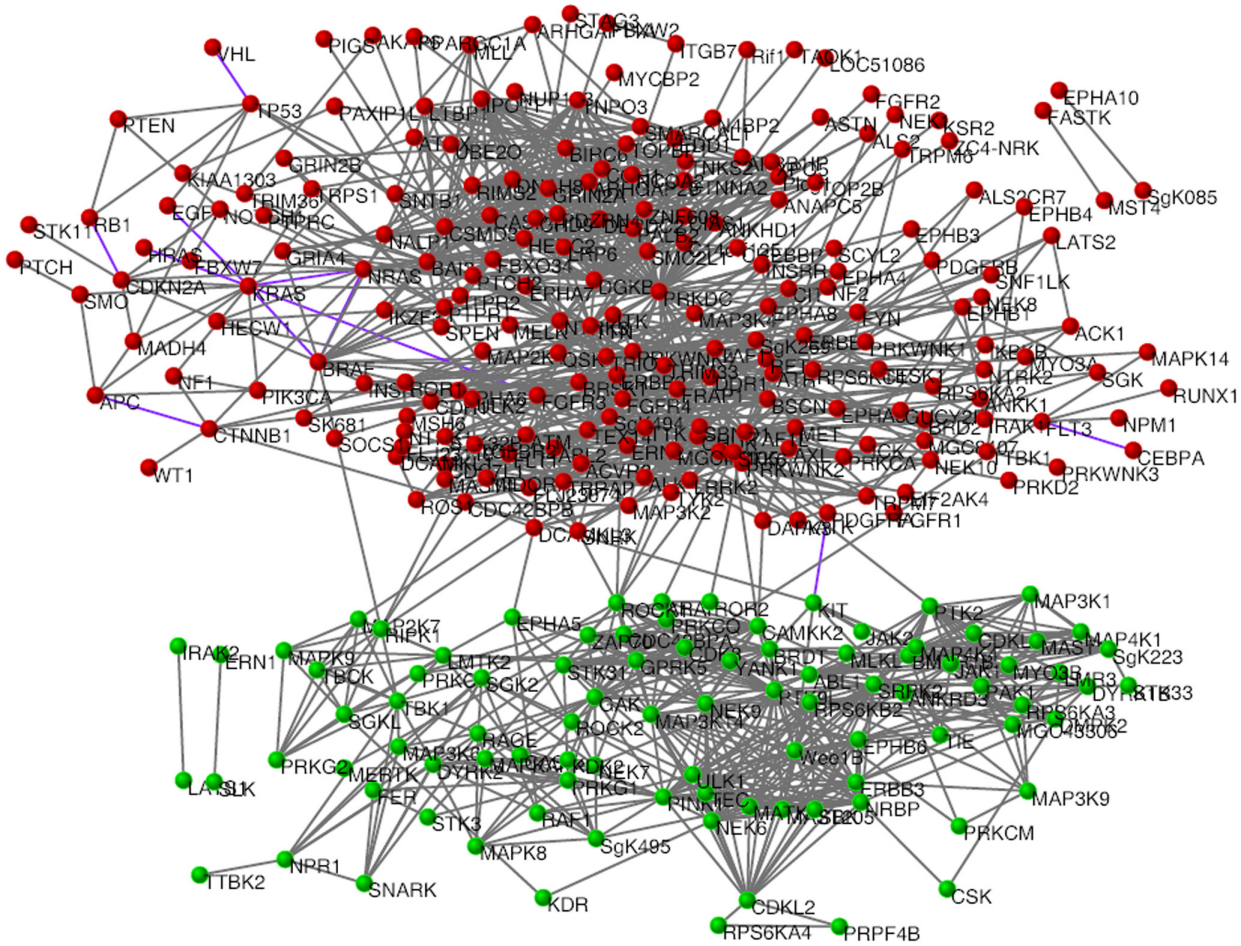
Network of cancer genes with co-occurring and anti-co-occurring mutations (CCA network). Each node represents one cancer gene. Genes connected by gray links represent co-occurring mutations. Genes connected by purple links represent anti-co-occurring mutations. The CCA network has two modules. Nodes in module one are red and nodes in module two are green.

A network of cancer genes with co-occurring and anti-co-occurring mutations (CCA network) was constructed by connecting cancer genes that have significant co-occurrence or anti-co-occurrence with other genes (see **Materials and methods**). The CCA network includes 306 genes and 1,366 links (File S1; Figure 1). Among the 1,366 links, 1,355 (99.2%) are links of co-occurring gene mutation and only 11 (0.8%) are links of anti-co-occurring gene mutation. Thus, in this study, all analyses were performed for genes with co-occurring mutations unless otherwise stated or explained. The CCA network includes five network components. The giant (biggest) network component contains 97.4% (298/306) of the total nodes, suggesting that most of the genes have potential relationships that contribute to tumorigenesis. The degrees of nodes in the CCA network show a distribution of power law (Figure S1), indicating that the CCA network is a scale-free network.

### Preference of genes with co-occurring mutations for direct activation signaling transductions

**Figure 2:**
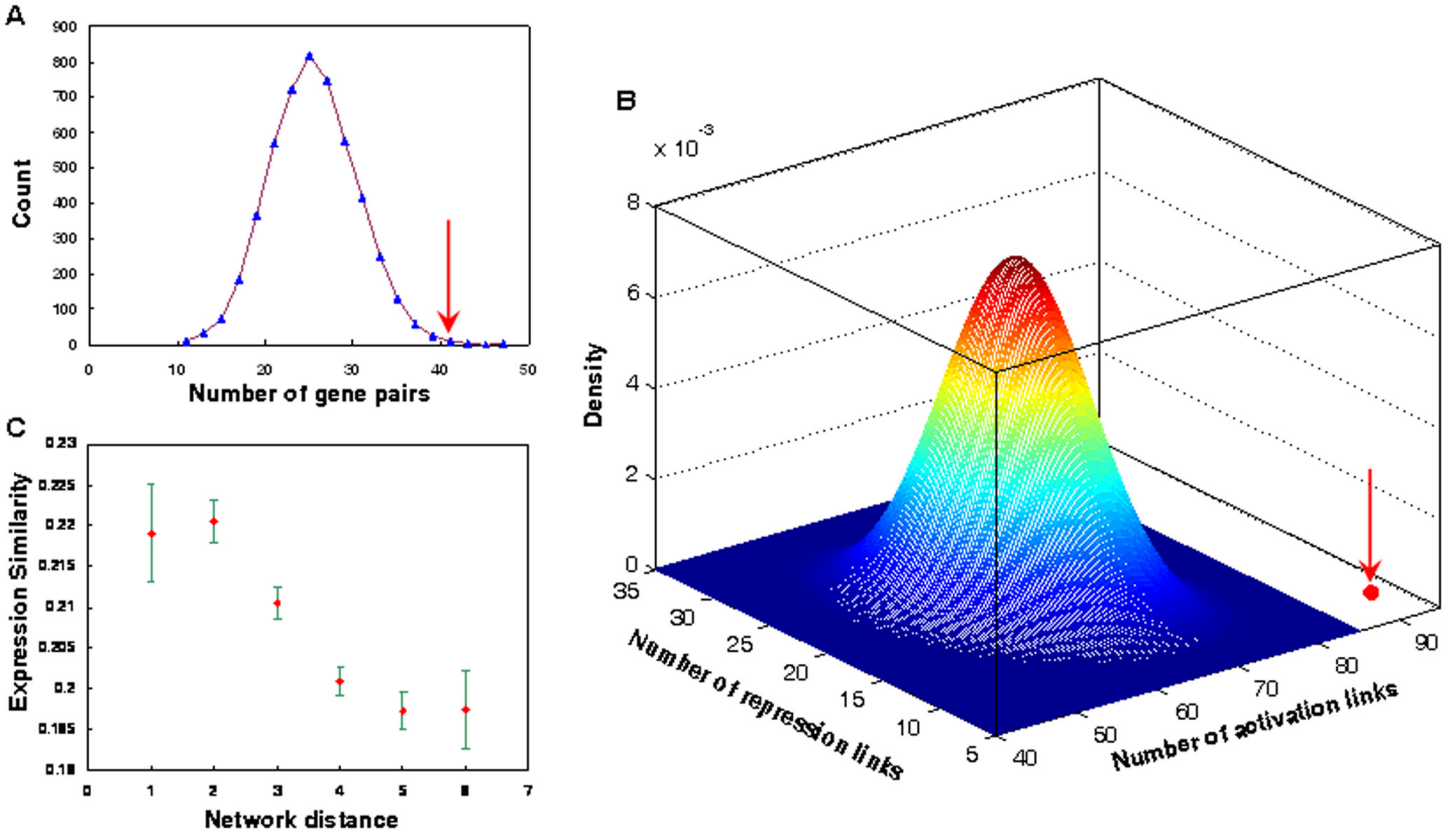
(A) Significance of cancer genes with co-occurring mutations existing in the same signaling pathways. The red arrow indicates the real number of cancer gene pairs that exist in the same pathway. The curve is the distribution of the random number of cancer gene pairs that exist in the same pathway. (**B**) Distribution of number of activation and repression signals among cancer gene pairs with co-occurring mutations. The red circle (pointed by a red arrow) indicates the real number of activation signals and repression signals that exist among the cancer gene pairs in the human signaling network. The heatmap indicates the union distribution of the random number of activation signals and repression signals that exist among the cancer gene pairs in the human signaling network. (**C**) Correlation between the distance of cancer genes in the CCA network and their expression similarity.

It is believed that cancer genes with co-occurring mutations are not randomly distributed in the cancer genome. Currently, it remains unclear how co-occurring mutations occur. As cells need to respond to various signals, and since cell signaling is critical to tasks such as cell growth, maintenance of cell survival, proliferation, differentiation, development, and apoptosis, the dysfunction of cell signaling from gene mutations can result in cancer[1]. To address the questions above, the distribution of genes with co-occurring mutations in human cell signaling pathways and in a human cellular signaling network was investigated. Co-occurring mutated genes were first mapped into 183 human signaling pathways, and 42 pairs of genes with co-occurring mutations were mapped to the same pathways. A randomization test was then performed to evaluate whether gene co-occurring mutations prefer to occur in the same signaling pathway (see **Materials and methods**). The results suggest that gene co-occurring mutations prefer to exist in the same signaling pathway (P = 0.002, randomization test, Figure 2A). This observation further suggests that mutations of a single gene in one signaling pathway may not necessarily lethally affect the pathway, and that dysfunctions of one pathway are often the result of the co-occurrence of mutations of two or more genes. To determine whether genes with co-occurring mutations tend to have direct interactions during cellular signaling, the distribution of cancer genes with co-occurring mutations in a human cellular signaling network was investigated. A total of 306 cancer genes were first mapped into the signaling network, and 107 of these genes were determined to exist in the signaling network. For pairs of genes with co-occurring mutations, 7.0% of them had direct interactions in the signaling network. In comparison, for the pairs of genes that did not have co-occurring mutations, only 2.8% of them had direct interactions in the signaling network. This indicates that genes with co-occurring mutations tend to have direct signaling interactions (P = 0.02, Fisher's exact test, odds ratio (OR) = 2.45). Cells must use correct signals (i.e. activation or repression) to make right response to various stimulus [14]. Different signals may lead cells to different fates [15], [16]. It is interesting to reveal which signals these interacting cancer genes prefer to use. For doing so, the number of different signals among the interacting cancer gene pairs was counted. The results show that these genes use more activation signals (69.2%), less repression signals (6.8%) and physical interactions (24.0%) than the average (47.5%, 14.6%, and 37.9% for activation, repression, and physical links, respectively). The signals used by these cancer genes are strongly unbalanced (P = 4.1×10^−6^, Chi-square test). Moreover, these cancer genes prefer to use activation signals and avoid using repression signals (P<2.0×10^−4^, randomization test; Figure 2B). These results suggest that, in many cases, mutations in a single gene in one signaling pathway cannot significantly affect cellular signaling, and that accumulated mutations in the activation signaling interaction tend to amplify the effects of cancer gene mutations and therefore have significant influences on the dysfunction of the signaling pathway. These mutations may therefore contribute more to cancer formation and development than other mutations.

### Network distance of genes in the CCA network correlates with their expression similarity

The above results suggest that cancer genes with co-occurring mutations tend to be more functionally related than random gene pairs. Therefore, genes that are close to each other in the network may be more functionally related than genes that are far away from each other. It has been reported that gene expression similarity has a good correlation with gene functional similarity[17], [18], [19]. Therefore, the network distance (the length of shortest path between two nodes in a network) of these cancer genes is expected to be correlated with their expression similarity. To confirm this expectation, the author first calculated the expression similarity of pairs of interacting genes in the CCA network based on human gene expression data presented by Su et al. [20] by Pearson's correlation, which is frequently used to calculate gene expression similarity (see **Materials and methods**). Next, the correlation between the network distance and expression similarity of genes in the CCA network was analyzed. As expected, network distance and expression similarity are negatively correlated (R = −0.04, P = 6.9×10^−11^, Spearman's correlation). Considering that many gene pairs have the same network distance, which may generate bias in the correlation analysis, gene pairs were further integrated into groups according to their network distance, and the average expression similarity for each group of gene pairs was calculated. As shown in Figure 2C, the network distance of grouped genes is negatively correlated with their expression similarity (R = −0.89, P = 0.03, Spearman's correlation). These results indicate that genes with co-occurring mutations tend to be more functionally related, and vice versa.

### Genes with co-occurring mutations tend to have similar mutation frequencies, whereas genes with anti-co-occurring mutations tend to have different mutation frequencies

**Figure 3:**
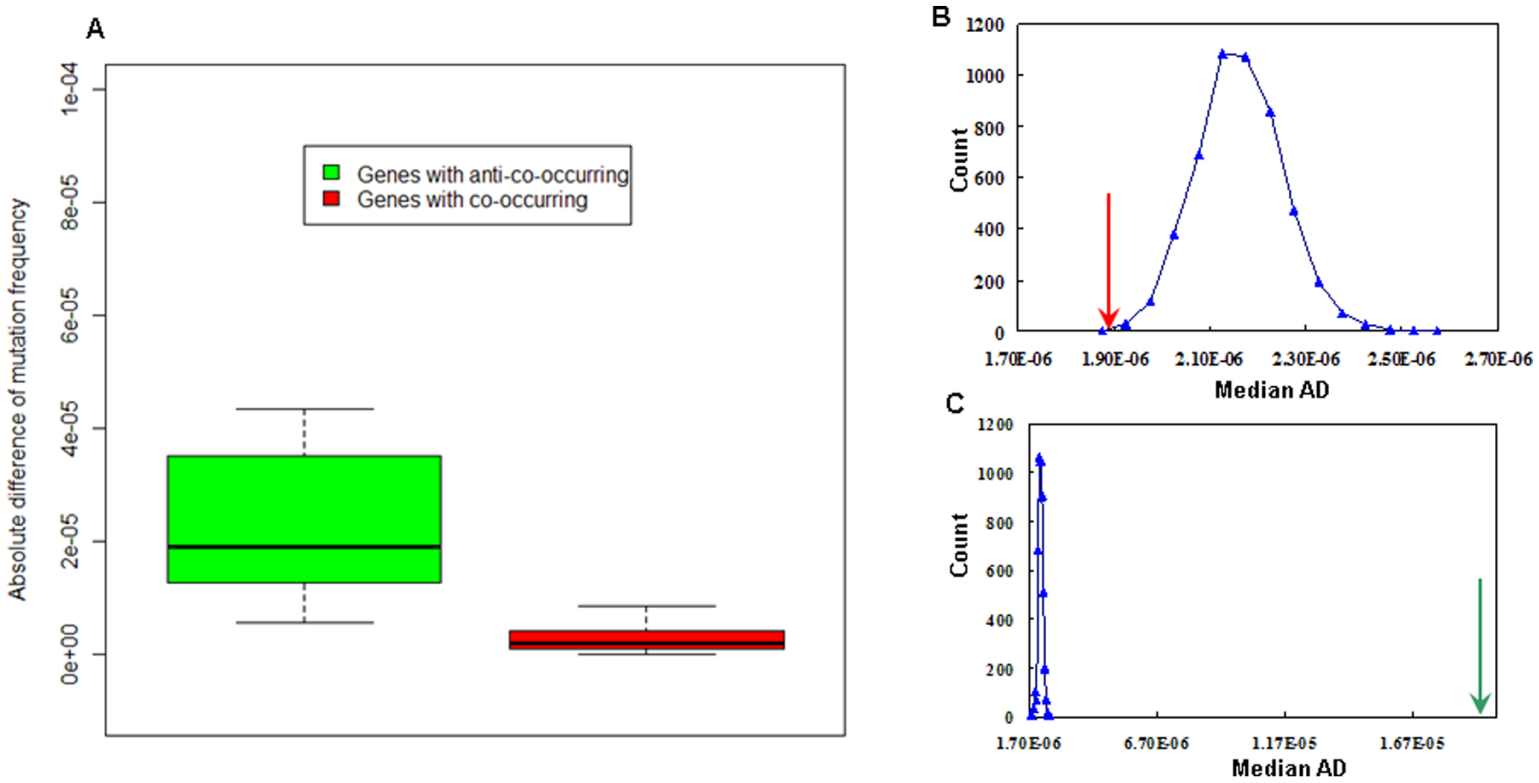
Comparison of absolute differences of mutation frequency (AD) among genes with co-occurring mutations, genes with anti-co-occurring mutations, and random gene pairs. (**A**) Comparison of AD values for genes with co-occurring mutations (red) and genes with anti-co-occurring mutations (green). (**B**) Comparison of median AD values for genes with co-occurring mutations (red arrow) and the distribution of the median values for 5,000 random groups of genes with co-occurring mutations. (**C**) Comparison of median AD values for genes with anti-co-occurring mutations (green arrow) and the distribution of the median AD values for 5,000 random groups of genes with anti-co-occurring mutations.

Mutation frequency varies for different cancer genes and different cancer types. For example, P53 is mutated in nearly 90% of oesophagus cancer samples but only in 7.5% of kidney cancer samples. Although a large scale of co-occurring mutated genes have been identified in this study, the patterns of the mutation frequency of these genes remain unknown. To address lack, the absolute difference of mutation frequency (AD) for any two neighbor genes in the network was calculated. The AD values for genes with co-occurring mutations, genes with anti-co-occurring mutations, and random gene pairs were then compared. Genes with co-occurring mutations were found to have smaller AD values than genes with anti-co-occurring mutations (P = 3.32×10^−7^, Wilcoxon test, Figure 3A). It was also found that genes with co-occurring mutations have smaller AD values than random gene pairs (P = 0.002, randomization test, Figure 3B), whereas genes with anti-co-occurring mutations have greater AD values than random gene pairs (P<0.0002, randomization test, Figure 3C).

### Network features are associated with gene features

An important characteristic of a node in a network is its degree[21]. The degree of a node is represented as the number of links it has. This is a vital metric for measuring the centrality of a node in a network. However, it remains unknown which genes tend to have more co-occurring mutations than other genes in the CCA network. As such, the associations of node degree with node length, mutation frequency, and number of exons were further investigated. Analysis results show that degree is not associated with mutation frequency or gene length but is positively correlated with the number of exons (R = 0.13, P = 0.02, Spearman's correlation). This suggests that proteins composed of more exons may have greater functional diversity and, therefore, may potentially have more physical or biochemical interactions with other proteins. This further suggests that co-occurrence of mutations of these proteins with other proteins may often be necessary for the dysfunction of various pathways that contribute to tumorigenesis.

The clustering coefficient (CC) is yet another important characteristic for nodes in networks [21] as it represents the coherence of local regions in networks. The CCA network has an average CC of 0.48. This indicates that mutations of multiple genes are often needed in tumorigenesis. Moreover, the CC of genes is negatively correlated with their mutation frequency (R = −0.19, P = 7.09×10^−4^, Spearman's correlation) and positively correlated with their degree (R = 0.19, P = 6.29×10^−4^, Spearman's correlation). Considering that genes with co-occurring mutations tend to be functionally related, as revealed above, this result suggests that genes in a densely interacted local network structure (i.e., genes with many mutual interactions) tend to be more robust against errors (e.g., mutations) and have smaller lethality than those in a sparsely interacted local network structure. Furthermore, both degree and CC increase from extracellular space to nucleus (median degrees are 3, 6, 6, and 6.5; median CCs are 0.17, 0.40, 0.44, and 0.45), indicating that downstream cellular components tend to cooperate more with each other in the cellular dysfunctions contributing to cancer than upstream cellular components.

### The CCA network shows modular structures

**Figure 4:**
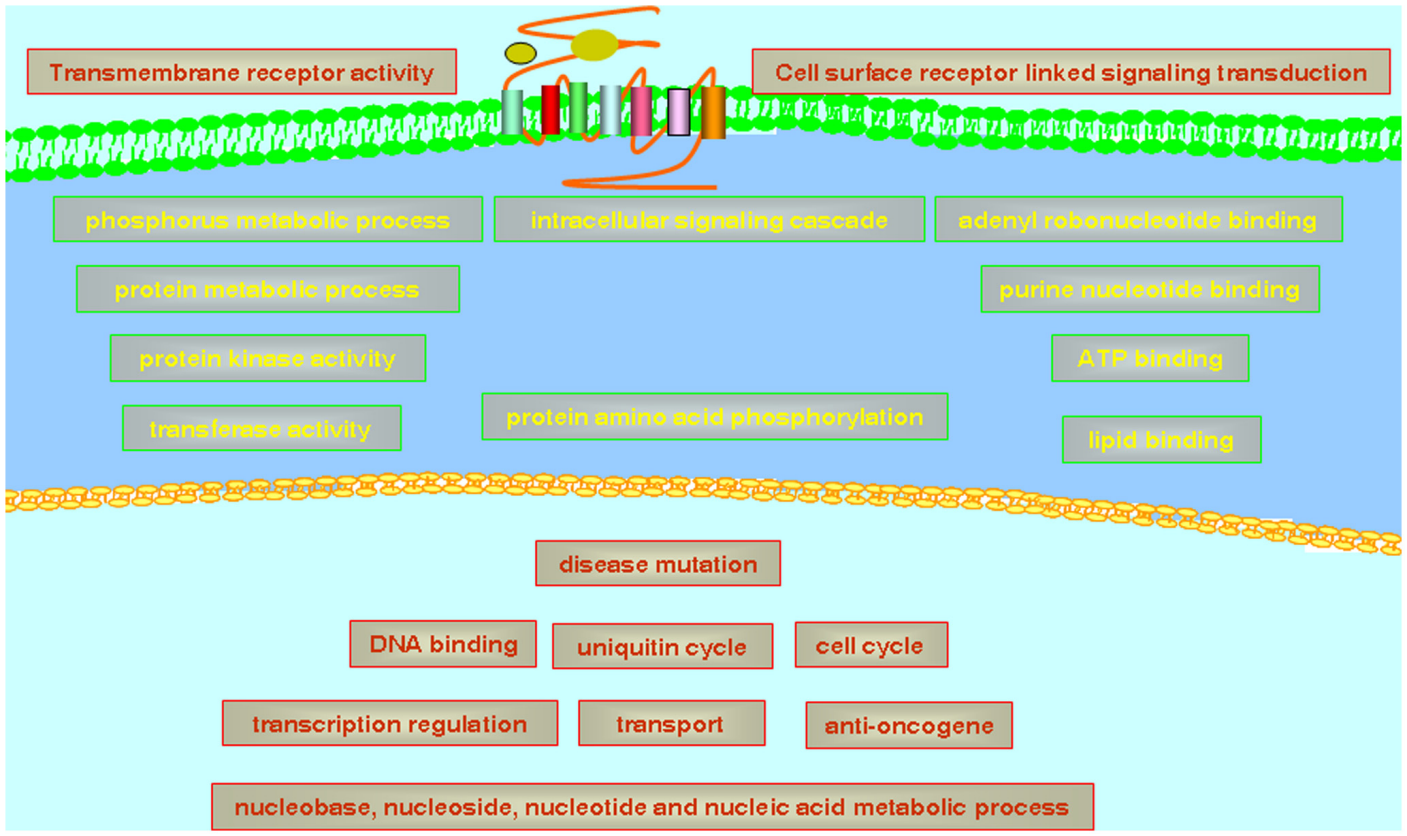
Cellular illustration of the enriched cellular components, molecular functions and biological processes of Module One and Module Two obtained by DAVID Bioinformatics [**23**]. Module One (words with red color) is enriched in the membrane and nucleus and Module Two (words with yellow color) is enriched in the intracellular space. The enriched functions and biological processes of each module are also plotted in the corresponding locations.

Similar to many other biological networks, the CCA network also shows modular structures. In a network, a network module refers to a highly interconnected group of nodes[21]. Nodes between two network modules are sparsely connected. As shown in Figure 1, the CCA network clearly has two modules. Previous studies have revealed that nodes in a network module tend to work together to achieve a specific function [21], [22]. To confirm whether or not the two modules in the CCA network have distinct functions, the enriched molecular functions (MFs), biological processes (BPs), and cellular components (CCs) for genes in the two modules were identified using the DAVID software [23] and setting all cancer genes in the two modules as the background population. DAVID is a popular tool to identify enriched gene sets (i.e. pathways, gene ontology terms) by evaluating the significance of enrichment of interested genes in each predefined gene set[23]. We took the gene sets with P values less than or equal to 0.05 as significant gene sets. The results reveal that the two modules have distinct enriched gene sets (Figure 4; File S2). In terms of cellular location, for example, Module One is enriched in the membrane and nucleus, whereas Module Two is enriched in the intracellular space. Both modules are enriched with the function of binding but tend to bind with different molecules. Module One is enriched with DNA binding, whereas Module Two is enriched with binding small molecules such as adenyl ribonucleotide, purine nucleotide, ATP, and lipids. Moreover, the two modules are also different in their metabolic and signaling processes. These results indicate that genes with co-occurring and anti-co-occurring mutations show specific architecture with regard to both topology and function.

**Figure 5:**
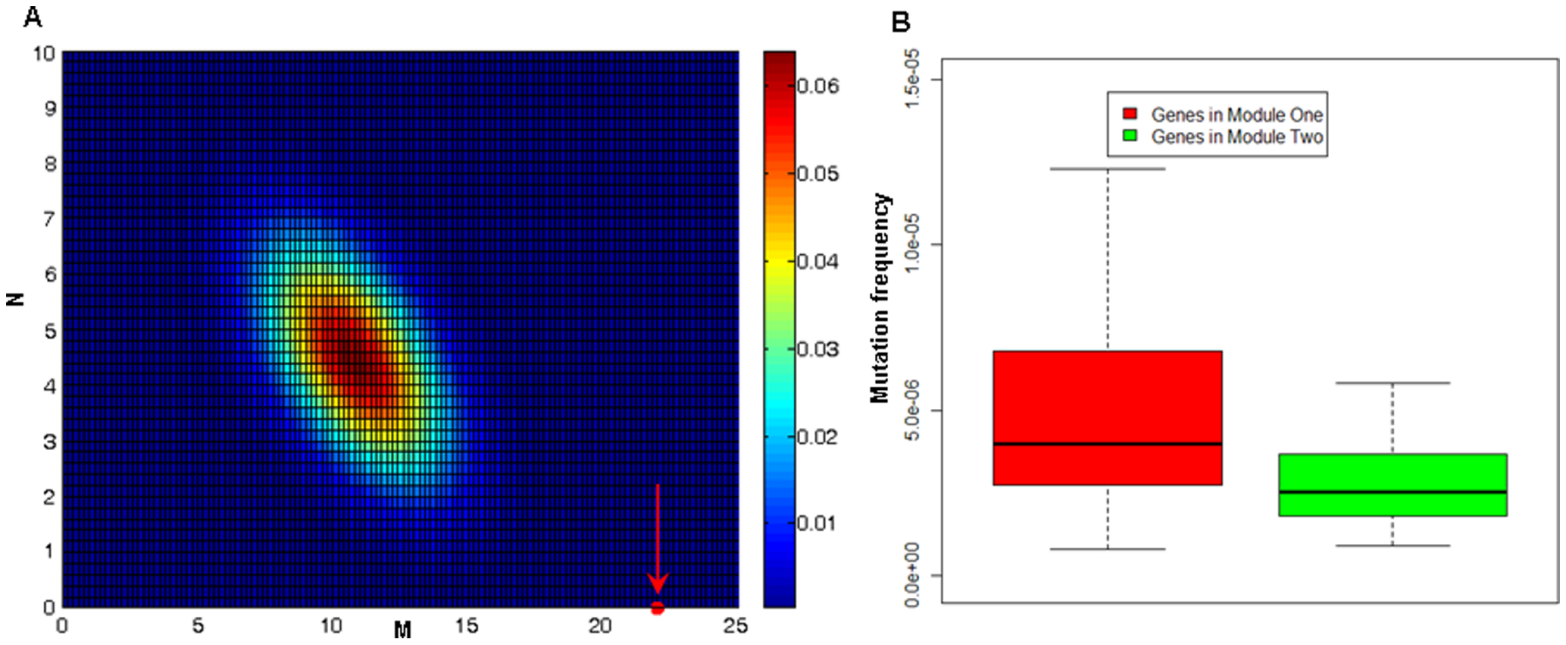
(A) Distribution of cancer gene mutations of the 22 cancer samples from Sjoblom et al. ' study [**7**] in two modules of the CCA network. The x axis represents the number (M) of samples that have at least one mutation in Module Two. The y axis represents the number (N) of samples that have mutations only in Module Two. The red circle (pointed by a red arrow) indicates the real number of M and N. The heatmap indicates the union distribution of the random number of M and N and its color represents the probability density. (B) Comparison of mutation frequency for genes in Module One and genes in Module Two.

The author asked if the genes in two modules could operate in a compensatory or concerted manner to govern various functions. Toward this end, an independent dataset of whole-genome cancer gene mutations of 22 cancer samples from Sjoblom et al. ' study [7] was used to investigate the distribution of gene mutations in the two modules. The author counted the numbers of samples that have gene mutations in both modules, only in Module One, and only in Module Two, respectively. As a result, among the total 22 cancer samples, 8 samples have gene mutations in both modules, 14 samples have gene mutations only in Module One, and no samples have gene mutations only in Module Two. Random modeling of the distribution of the three numbers shows that samples have gene mutations in both modules occur with a chance of 25% in random case. This result suggests that genes from the two modules have a tendency to work together in a complementary way to generate tumor phenotypes. Moreover, all samples have at least one gene mutation in Module One. This number is significantly greater than the random case (P<2.0×10^−4^, randomization test; Figure 5A), indicating that this module seems to be the critical and essential player in tumorigenesis. This finding is further supported by the following observations. (a) Module One is enriched in function of anti-oncogene (File S2). (b) Genes in Module One have higher fraction of methylation (10.0%, 21/210) in cancer stem cells than those in Module Two (1.1%, 1/88). The author obtained the numbers of methylated genes in these two modules by first mapping methylated genes into genes in these two modules and then counting the numbers of mapped methylated genes in Module One and Module Two, respectively. Fisher's exact test shows that cancer methylated genes has significantly enriched distribution in Module One (P = 0.006), suggesting that genes in Module One contribute more to the long-term loss of gene expression and represents the early stage of tumor formation[1]. (c) The mutation frequency of genes in Module One is significantly higher than that of genes in Module Two (P = 5.75×10^−9^, Wilcoxon test; Figure 5B). The statistical significance was obtained using Wilcoxon test to test the difference between two groups of cancer gene mutation frequency from Module One and Module Two.

## Discussion

Although a number of cancer genes contribute to tumorigenesis in a manner of co-occurring or anti-co-occurring mutations, it has been challenging to gain a global landscape of when, where, and how their interactions exert affects on tumor formation and development. By building a CCA network and performing an integrative analysis of this network, the author uncovered a global picture of cancer gene co-occurring and anti-co-occurring mutations. Co-occurring mutations preferentially occur in functionally related gene pairs, such as signaling transduction molecules, especially the activation signaling transduction molecules. The cancer gene interacting pairs have an affect on their mutation frequencies. Genes with co-occurring mutations tend to have similar mutation frequencies, whereas genes with anti-co-occurring mutations tend to have different mutation frequencies. Topologically, the CCA network shows two modules that have specific functions. The two modules tend to have functional collaborations and Module One seems to be the central player in contributing to tumorigenesis.

Because of the tremendous diversity and complexity of cancer gene mutations, cancers even the same type of cancers show very different mutation profiles and few common patterns behind these data have been identified. Therefore, some researchers doubt the value of cancer genomics [24]. By a systems level analysis, this study has presented direct evidence for the principles of cancer gene co-occurring and anti-co-occurring mutations, which are helpful in understanding the mechanism by which cancer genes contribute to cancer formation and development in a manner of co-occurring or anti-co-occurring mutations.

The sequencing of cancer genomes is an ongoing process. Currently, the sequenced mutation data can interpret only a portion of the mutations. As the development of next generation genome sequencing technology, more and more cancer gene mutation data will be accumulated. Therefore, it is expected that more reliable and novel observations will be realized in the future. This study presents a systems biology framework for the integrative analysis of cancer genome sequencing outputs, which will be useful for not only the understanding of tumorigenesis but also the identification of valuable biomarkers and drug targets.

## Materials and Methods

### Cancer gene mutation data

Cancer gene mutation data were downloaded from the Catalogue of Somatic Mutations in Cancer (December 2008, COSMIC, http://www.sanger.ac.uk/genetics/CGP/cosmic/). The mutation frequency of a gene was calculated by dividing the number of total cancer samples that sequenced this gene with that of cancer samples with at least one mutation of this gene, as described by Cui et al. [1]. Because gene mutation frequency is significantly correlated with gene length (R = 0.4, P = 5.16×10^−14^, Spearman's correlation), gene mutation frequency was further corrected by gene length using formula “original mutation frequency/gene length”.

### Methylated genes in cancer stem cells

The 287 methylated genes in cancer stem cells were obtained from Cui et al' s study [1], in which they collected these genes from three studies[25], [26], [27]. Twenty two of the 287 genes were found on the CCA network.

### Human signaling pathway and cellular signaling network

One hundred and eighty four human signaling pathways were obtained from BioCarta (http://www.biocarta.com/) and a human cellular signaling network was obtained from the study of Cui et al.[1]. The human cellular signaling network included 1,634 nodes and 5,089 links, containing 2,403 activation interactions, 741 repression interactions, 1,915 physical interactions, and 30 links whose types are unknown (File S3).

### Network of co-occurring and anti-co-occurring cancer gene mutations

For any two genes, for example, A and B, the number of cancer samples that sequenced both Gene A and Gene B was first identified. The number of cancer samples with mutations in both Genes A and B (AandB), that of cancer samples with mutations only in Gene A (AnotB), that of cancer samples with mutations only in Gene B (BnotA), and that of cancer samples with mutations in neither Gene A nor gene B (notAnotB) were then counted. The significance (P value) of co-occurring or anti-co-occurring cancer gene mutations was further determined using Fisher's exact test based on the obtained four numbers. A network of co-occurring and anti-co-occurring cancer gene mutations (CCA network, File S1) was constructed by setting a P value cutoff 0.02. Any two genes with P value less than or equal to 0.02 (FDR P< = 0.08) were linked. Finally, a CCA network was constructed from this method. The final network included 306 nodes and 1,366 links (Figure 1), which was drawn by pajek, a free network visualization and analysis software (http://vlado.fmf.uni-lj.si/pub/networks/pajek/).

The degree and CC value for each node in the CCA network was calculated according to the formula presented by Barabasi and Oltvai (File S4) [21]. The mutation frequency of cancer genes is also given in File S4. The network components were identified by a Java program designed for this purpose (File S5). The network distance of two genes was obtained by calculating the length of their shortest path, which was implemented using Dijkstra's algorithm. The degree distribution was calculated by R, a free statistical software (http://cran.r-project.org/). As the CCA network showed very clear modular structures, two network modules (File S6) were manually identified without using other tools.

### Analysis of gene expression data

This study obtained the normalized human gene expression profile across 79 human tissues from Su et al. ' study [20] for the analysis of gene expression similarity. The author extracted expression profiles for genes included in the CCA network and further measured the expression similarity of any two linked genes in the CCA network by absolute Pearson's correlation coefficient, which is a frequently used metric in similar analysis [17].

### Statistical computations

All statistical tests were performed using R software. The randomization tests were performed by Java programs designed for this purpose. The basic idea of the randomization tests was introduced here by taking the test for the enrichment of co-occurring mutations in human signaling pathways as an example. Genes were first randomly re-connected in the network using the same number of links. The number of re-generated links that are within the same signaling pathways was then counted. These procedures were repeated 5,000 times. The number of times (T) the random number was greater than or equal to the real number was counted. The P value was calculated using the formula *(T+1)/5001*.

## Supporting Information

File S1 The pajek network file of the network of cancer genes with co-occurring and anti-co-occurring mutations
(0.05 MB TXT)

File S2 List of enriched molecular functions (MFs), biological processes (BPs), and cellular components (CCs) of genes in module 1 and module 2.
(0.05 MB DOC)

File S3 List of the genes, their functions, cellular locations and signaling relations in the human signaling network.
(0.49 MB XLS)

File S4 List of network nodes and their mutation frequency, degree and clustering coefficient (CC).
(0.01 MB TXT)

File S5 The jave source code file for the identification of network components.
(0.00 MB TXT)

File S6 List of genes in two network modules.
(0.00 MB TXT)

Figure S1 Degree distribution of the CCA network.
(0.41 MB TIF)
