# Supplementary material for: A Network of Cancer Genes with Co-Occurring and Anti-Co-Occurring Mutations
Source: PLoS One. 2010 Oct 4;5(10):e13180. doi: 10.1371/journal.pone.0013180 (PMC2949398; doi:10.1371/journal.pone.0013180)
Supplement: File S2 — List of enriched molecular functions (MFs), biological processes (BPs), and cellular components (CCs) of genes in module 1 and module 2. (0.05 MB DOC) [file pone.0013180.s002.doc]

File S2. List of enriched molecular functions (MFs), biological processes (BPs), and cellular components (CCs) of genes in module 1 and module 2.

Module 1

| Category | Description | P value |
| --- | --- | --- |
| MFs | DNA binding | 6.9e-4 |
|  | Transcription regulation | 3.6e-2 |
|  | anti-oncogene | 5.0e-2 |
|  | transmembrane receptor activity | 4.2e-2 |
| BPs | nucleobase, nucleoside, nucleotide and nucleic acid metabolic process | 3.0e-2 |
|  | regulation of cellular metabolic process | 4.9e-2 |
|  | cell cycle | 2.9e-2 |
|  | disease mutation | 4.0e-2 |
|  | negative regulation of metabolic process | 1.8e-2 |
|  | transport | 1.2e-3 |
|  | establishment of localization | 2.9e-3 |
|  | cell surface receptor linked signal transduction | 6.4e-3 |
|  | ubiquitin cycle | 4.9e-2 |
| CCs | membrane | 4.8e-2 |
|  | nucleus | 3.1e-2 |

Module 2

| Category | Description | P value |
| --- | --- | --- |
| MFs | transferase activity | 6.4e-14 |
|  | adenyl ribonucleotide binding | 1.8e-13 |
|  | purine nucleotide binding | 4.9e-13 |
|  | ATP binding | 1.8e-13 |
|  | lipid binding | 4.4e-2 |
|  | protein kinase activity | 2.7e-15 |
| BPs | phosphorus metabolic process | 6.5e-13 |
|  | protein metabolic process | 4.8e-8 |
|  | intracellular signaling cascade | 4.3e-3 |
| CCs | cytoplasm | 5.3e-2 |
