# Supplementary figures and images for: A Network of Cancer Genes with Co-Occurring and Anti-Co-Occurring Mutations
Source: PLoS One. 2010 Oct 4;5(10):e13180. doi: 10.1371/journal.pone.0013180 (PMC2949398; doi:10.1371/journal.pone.0013180)

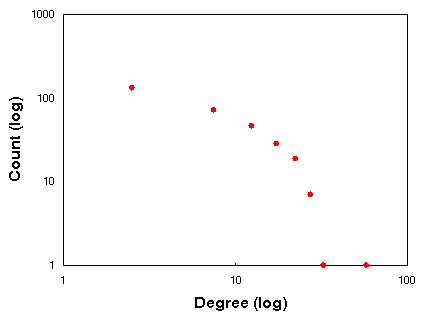

Supplement: Figure S1 — Degree distribution of the CCA network. (0.41 MB TIF) [file pone.0013180.s007.tif]
